# **Pea** Albumin 1 Subunit b (PA1b), a Promising Bioinsecticide of Plant Origin

**DOI:** 10.3390/toxins3121502

**Published:** 2011-12-08

**Authors:** Frédéric Gressent, Pedro Da Silva, Vanessa Eyraud, Lamis Karaki, Corinne Royer

**Affiliations:** INSA-Lyon, INRA, Université de Lyon, UMR203 BF2I, Biologie Fonctionnelle Insectes et Interactions, Bat. Louis-Pasteur 20 av. Albert Einstein, Villeurbanne F-69621, France; Email: Pedro.da-silva@insa-lyon.fr (P.D.S.); Vanessa.Eyraud@lyon.inra.fr (V.E.); Lamis.Karaki@lyon.inra.fr (L.K.); Corinne.Royer@lyon.inra.fr (C.R.)

**Keywords:** PA1b, insect, bioinsecticide, Legumes

## Abstract

PA1b (Pea Albumin 1, subunit b) is a peptide extract from pea seeds showing significant insecticidal activity against certain insects, such as cereal weevils (genus *Sitophilus*), the mosquitoes *Culex pipiens* and *Aedes aegyptii,* and certain species of aphids. PA1b has great potential for use on an industrial scale and for use in organic farming: it is extracted from a common plant; it is a peptide (and therefore suitable for transgenic applications); it can withstand many steps of extraction and purification without losing its activity; and it is present in a seed regularly consumed by humans and mammals without any known toxicity or allergenicity. The potential of this peptide to limit pest damage has stimulated research concerning its host range, its mechanism of action, its three-dimensional structure, the natural diversity of PA1b and its structure-function relationships.

## 1. Introduction

Chemical pesticides in general, and insecticides in particular, are increasingly used around the world, but they are also increasingly stigmatized because of their persistence and their toxicity to non-target organisms (impacting amphibians, aquatic wildlife, beneficial insects such as bees and ladybugs, and even causing mortality among farmers, particularly in developing countries). Crop protection against two very important pests, namely cereal weevils and aphids, is currently carried out almost exclusively by chemical treatments. A few alternative methods do exist to combat these insects, but they are either much less effective or prohibitively expensive, compared with chemical control. 

Chemical treatments used to protect stored products are the source of the majority of chemical residues in cereals which are subsequently found in processed products. These residues can be dangerous for consumers if present in high doses. However, the presence of insects or mites is the most common cause of refusal of grain deliveries to the food industry due to non-compliance with health regulations. 

Hence, it is imperative to find new molecules with less impact on the environment. One of the most promising sources for such compounds is probably plants, which have developed many ways to fight against insects (as well as against fungal and bacterial attacks) and one of these is the use of chemicals. Insecticidal molecules are found in other organisms, such as spiders or scorpion venom, but then they are generally toxic for mammals as well. The search for entomotoxic components in plants, and specifically in plants that are consumed by mammals, could be a valuable approach in order to develop biopesticides for sustainable and healthy agriculture.

Numerous molecules have yet to be identified in plants as regards their ability to counter insect, fungal or bacterial attacks. These molecules could be of a proteinaceous nature, including thionins, defensins, lipid transfer protein, snakins or protease inhibitors. They may also result from the secondary metabolism of plants, and many defense molecules are of an alkaloid, saponin or flavonoid nature (for review see [1]). 

Amongst these, one of the most promising molecules is the PA1b (Pea Albumin 1, subunit b) peptide, extracted from Legume seeds. The presence of a toxic compound in peas was first suspected by Delobel *et al.* [xref^2^]. Purification of the lethal molecule led to the identification of a peptide [3] whose primary structure and gene structure was elucidated, in 1986, by Higgins *et al.* [xref^4^], though without any demonstration of its toxic effects. PA1b is one of the few orally active entomotoxin peptides currently known. It displays outstanding insecticidal activity against certain insects, such as grain weevils (genus *Sitophilus*) and some species of aphids, and this insecticidal activity has been patented [3]. PA1b is a peptide that has many attributes for use on an industrial scale: it is extracted from a commonly grown plant; it is a peptide (suitable for use in transgenic plants); and it can withstand many stages of extraction and purification without losing its activity. PA1b also has many advantages in terms of agricultural use. It is a peptide purified from a plant (*Pisum sativum*) and, moreover, a plant regularly consumed by humans and other mammals without any sign of toxicity or allergenicity. 

## 2. PA1b Sequence and Properties

**Figure 1:**
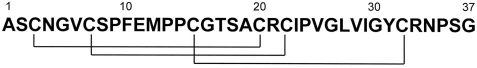
PA1b primary structure. The cysteine residue pairing is shown below [3].

PA1b is a peptide consisting of 37 amino acids, including six cysteines involved in three intramolecular disulfide bridges (Figure 1). The three-dimensional structure of the peptide has revealed that it belongs to the cystine knot inhibitor (ICK) family ([5], see Section 6). This folding confers a very high level of resistance: the PA1b peptide maintains its biological activity after boiling [3], following extraction by different solvents or solutions (methanol, pentane, ether acetate, acetone, 15% TCA in water or 5% NH3 in water, after digestion by proteases (including trypsin, papain and proteinase K, with the exception of pronase E) or digestion by the gut extract of insect or rumen degradation [6,7,8]. These characteristics also permit a simple purification process of the PA1b peptide, from pea flour, by solvent extraction and classical reverse-phase HPLC [3]. 

These features, together with the potential of this peptide to limit pest damage, has generated considerable research into its host range, its mechanism of action, its three-dimensional structure, the natural diversity of PA1b and its structure-function relationship. 

## 3. Toxicity Host Range of PA1b

### 3.1. Biological Activity on Insects

PA1b was first identified by its ability to kill cereal weevils (*Sitophilus oryzae*, *S. zeamais* and *S. granarius*). Several other insect species were tested for their susceptibility to the toxic peptide and it appears that not all insects are susceptible, unrelated to the taxonomy of the species tested. For example, out of three species of aphids, *Acyrthosiphon pisum* is sensitive, *Myzus persicae* is not sensitive and *Aphis gossypii* is only sensitive at high doses). The mosquitoes *Culex pipiens* [6] and *Aedes aegyptii* [9] are both highly sensitive which is, of course, of great interest for the potential use of PA1b (on an industrial scale) to control these human and mammalian disease vectors. Some beneficial insects, such as the bee (*Apis mellifera*) and trichogramma (*Trichogramma pretiosum*), are insensitive, but both the development and the survival of the lady beetle (*Harmonia axyridis*) are strongly affected by the toxin [6]. It seems that Coleoptera are generally sensitive (with the exception of *Tribolium castaneum*) whereas the Lepidoptera are mainly insensitive, with the exception of *Ephestia khuniella* [3]. Several agronomically important caterpillars (*Mamestra brassicae*) [6], *Spodotera littoralis* and *Ostrinia nubilalis* [10] are not affected by PA1b. However, the cultured cells Sf9, originating from *S. frugiperda*, displayed high sensitivity to PA1b at low doses [11], indicating that *S. frugiperda* has all the molecular equipment to trigger the PA1b signal, but that certain insects are able to curtail the toxic effects. 

A binding site for PA1b has been found in the weevil gut membranes [12] (see Section 4.1). This receptor is present in all insects, suggesting the existence of a mechanism of insensitivity independent of the receptor. This mechanism of resistance is still unclear, but it is not due to a differential degradation in the insect gut. Indeed, gut extract from the insensitive insect *M. brassicae* was unable to degrade PA1b [6], as was gut extract from the sensitive weevil [3]. However, *Bruchus pisorum* (the pea weevil) is a notable exception this being the only insect, among those tested, to feed on legume seeds and also the only one in which the receptor is undetectable in the gut extract. This data suggests that PA1b is the source of insect resistance in Legume seeds and, therefore, adaptation to feeding on Legume seeds necessitates a loss of binding capacity of the toxin [6].

### 3.2. Biological Activity on Other Living Organisms

Biological tests performed on mammalian cells [11], bacteria, fungi [6], yeast [13] and nematodes [14] have all shown that PA1b has no effect on these cells or organisms. 

However, two biological effects of PA1b have been described outside of the insect world. In plants (specifically a carrot cell culture), it has been found that cell proliferation is enhanced by addition of the soybean homolog of PA1b, leading the authors to suggest that PA1b could act as a new peptidic plant hormone [15] (see Section 4.2 for more details). In mammals, injections of PA1b in mice were found to have a strong hyperglycemic effect, suggesting that PA1b could interfere with glucose metabolism and, in particular, with insulin perception in mammals [16] (see Section 4.3 for more details). 

## 4. The Molecular Mechanism of PA1b Action

As detailed below, PA1b has physiological effects on insects, plants and mammals. Moreover, the state of research indicates that the peptide probably acts in all organisms via an interaction with a receptor, but the receptor is probably different in insects, in plants and in mammals. Thus, PA1b could present the possibility of three modes of action by way of three interacting proteins. The recent concept of peptide promiscuity, describing how a unique peptide structure may be associated with multiple functions, has also been developed to explain plant peptide defense [17]. In particular, molecules, such as defensins [18] and cyclotides [19], with structural motifs also found in PA1b (small peptide, 3-4 disulfide bridges, and even the knotted fold for cyclotides) are able to have multiple functions with reduced modifications in the primary structure and conservation of the tertiary structure. PA1b could be an example of this diversity of functions for a single structural motif, a property now demonstrated for other members of the cystine-knot peptide family [20]. 

### 4.1. PA1b Mechanism of Action in Insects

Because of the interesting biological characteristics of PA1b, the molecular mechanism of action and the potential mechanism of resistance existing in insects were amongst the first questions to be addressed. A screening of 90 strains of weevils, belonging to all three species, was used to select four strains, all belonging to the species *S. oryzae* and fully resistant to the purified toxin, showing that resistance could occur naturally in the absence of selection pressure. Genetic studies have shown that resistance was monogenic and recessive [21], suggesting that the toxicity of PA1b is manifested via a receptor within the insect. With the help of a toxin labeled with iodine 125 (^125^I-PA1b), a saturable, reversible and high affinity (affinity in the nanomolar range) proteinaceous binding site has been characterized in membrane extracts of a susceptible strain of *S. oryzae*. A similar binding capacity was found in all the sensitive *Sitophilus* strains, but no specific binding was detectable in the four resistant strains of *S. oryzae*. This specificity strongly suggests that the characterised binding site is the molecular target of PA1b, and that resistance in *S. oryzae* implies an absence or modification of this target [12].

PA1b belongs to the knottin family which contains peptides with various biological targets, including enzymes and ionic channels [22]. Moreover, the structural analyses have also revealed similarities between PA1b and the ICK atracotoxin ACTX-Hi:OB4219, isolated from the insectivorous Australian funnel-web spider *Hadronyche infensa*, which targets an unknown channel [5]. On this basis, electrophysiological studies have been performed using PA1b on Sf9 insect cultured cells. The electrophysiological results clearly show that the toxin depolarizes the membrane of Sf9 cells and a pharmacological study demonstrated that PA1b was able to reproduce the bafilomycin effect, bafilomycin being a fungal inhibitor of V-ATPase [23].

V-ATPase is a proton pump using ATP, first characterized in the vacuolar membranes, but we now know that there are also plasmalemmic forms. V-ATPase is a multimeric protein complex, highly conserved among living species from bacteria to humans. In insects, the protein from *Manduca sexta* (Lepidoptera) has been closely studied as regards its structure, function and regulation [24,25]. This is an essential protein for insects, especially in the gut, since its action provides the energy required for the absorption of nutrients [25,26]. The insect V-ATPase is composed of 14 subunits organized into two complexes. The membrane complex V_0_ has four subunits, including the subunits c and a which form the proton channel, and the V_1_ complex is cytosolic and bears the ATPase function [27]. Biochemical tests performed on purified V-ATPase from *M. sexta* have shown that PA1b inhibits the V_0_V_1_ enzyme but not the V1 complex alone; the authors concluded that PA1b acts by binding and inhibiting the V_0_ complex of V-ATPase in insects [23].

### 4.2. PA1b Activity and Mechanism of Action in Plants

In soybeans, a 43-kDa protein exists which was found to bind insulin and insulin-like growth factor. It was shown that a 4-kDa peptide extract from soybean binds this protein and could compete with insulin for binding. Moreover, the 4-kDa peptide is able to stimulate the phosphorylation activity of the 43-kDa protein. For these reasons, the peptide was named leginsulin and was found to be the soybean isoform of the pea peptide PA1b [28,29]. The expression of leginsulin in cultured carrot cells leads to an enhanced cell proliferation [15]. A glycoprotein homolog to the 43-kDa protein from soybean exists in carrots and binds both insulin and leginsulin [30,31]. Authors then suggested that leginsulin (and, by extension, PA1b) could behave in plants as a hormone-like peptide [15] and that it acts by interacting with the 43-kDa protein, thus generating a cell signal transduction via the phosphorylation activity of the 43-kDa protein. So, a receptor seems to exist in plants (and not only in Legumes, although PA1b has, to date, only been found in Legumes) but it appears to be different from the one present in insects. In fact, the amino acid residues involved in binding were not the same for the interaction with the plant receptor [32] and with the insect receptor [33] (see Section 6).

### 4.3. PA1b Activity and Mechanism of Action in Mammals

Recent data from the literature show that subcutaneous injections of aglycin in mice (10 µg/g body weight) causes a transient, but highly significant, increase in blood glucose (+100% in 60 min). In fact, PA1b, aglycin and leginsulin are homologs, and all isoforms of the pea peptide have the same effect on mice. Using surface plasmon resonance biosensor technology, a single aglycin binding protein with an apparent molecular mass of 34-kDa was detected in membrane protein extracts from porcine and mice pancreas. Using affinity chromatography, the protein has been purified and identified, by mass fingerprinting, as a voltage dependant anion-selective channel (VDAC-1), a chloride channel first found in mitochondrial membranes but now known to be present in plasma membranes [16,34]. Thus, the plant peptide could interact with the physiology of mammalian cells (but by injection, not by ingestion as in insects), and particularly with the metabolism of glucose. This effect may be mediated by a receptor different from those found in plants or insects, but a receptor which is, as in insects, an ionic target, the VDAC-1. Linked to the glucose metabolism, electrophysiological studies have been performed on pancreatic β-cells of rat. PA1b may increase the intracellular level of calcium, as does insulin, but using a different mechanism. PA1b seems to act via Ca^2+^ influx through L-type Ca^2+^ channels. As PA1b (leginsulin) could compete with insulin in the plant system, the authors evoke the possibility that PA1b binds to the mammalian insulin receptor [35]. In mammals, the action of PA1b on metabolism seems to be well demonstrated, but the mechanism involved still remains unclear. Whether PA1b acts through the interaction with VDAC-1, with the L-type Ca^2+^ channels or with the insulin receptor is still under investigation. However, in insects, insulin does not compete with PA1b for the receptor, indicating that the receptor in insects (V-ATPase) is different from the one in mammals.

## 5. PA1b Biodiversity

### 5.1. The Structure and Regulation of the PA1 Gene

**Figure 2:**
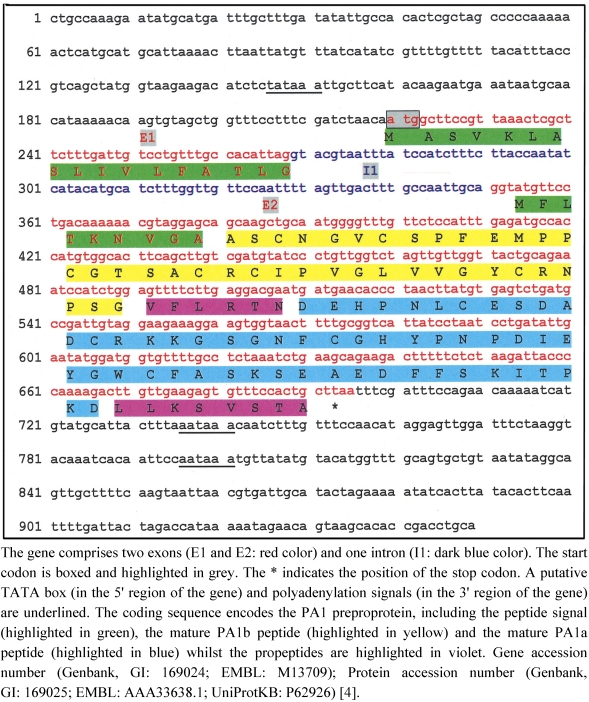
The gene and amino acid sequences of pea albumin 1 (PA1).

The structure of the pea albumin 1 reference gene (PA1 gene) is depicted in Figure 2. It was first described, in 1986, by Higgins et al. [4]. Two exons, separated by a short intron located inside the coding leader sequence, are present in the gene. The PA1 gene is transcribed as a single mRNA encoding the preproprotein PA1 [molecular weight 13.9 kDa; 130 amino-acids (aa)] which is co-translationally targeted to the endoplasmic reticulum (ER) by cleavage of its signal peptide (2.7 kDa, 26 aa). The proprotein precursor (11.2 kDa, 104 aa) is directed to the vacuole-like, seed-protein storage bodies where it is endoproteolytically cleaved to yield two peptides which, after removal of some carboxyl-terminal amino-acids (propeptides), represent the mature forms of PA1a (6 kDa, 53 aa) (*C*-terminal fragment) and PA1b (3.8 kDa, 37 aa) (*N*-terminal fragment) [4]. 

Although the PA1 gene has been used extensively in genetic engineering, paradoxically little is known about its regulation and, more specifically, upstream *cis* regulatory elements that could positively or negatively affect the rate of transcription have not yet been identified. However, computational analysis based on the increasing use of growing genomic resources (genome sequencing, EST databases *etc.*) should soon provide new data on the distribution and tissue-specific expression of PA1 homologous genes, as is already the case for the identification of novel genes including those encoding cysteine-rich peptides [36,37].

Some studies have provided evidence that the PA1 gene is regulated both spatially and temporally. In the pea, regulation of PA1 gene expression during the development of normal, non-deficient seeds appears to be under direct transcriptional control. There is little, or no, transcription of these genes in early development but then transcription progressively increases to reach its highest level midway through development (20 to 22 days after flowering), followed by a sharp decline [4,38]. In the genome of another legume, the narrow-leafed lupin (*Lupinus angustifolius*), a PA1b-like gene was detected that showed, within the same region, 96% sequence identity with the leginsulin gene from soybeans [39]. The gene was functional at the transcriptional level since the corresponding mRNAs were detected but they were in very low quantities and, almost exclusively, in developing seeds (mainly in cotyledons 40 days after anthesis) [39]. However, unlike in soybeans [29], no evidence was found for the expression of the leginsulin-like gene in the radicle of lupins (two days after germination), suggesting a tight temporal regulation of the gene in developing and germinating seeds [39].

An environmental regulation linked to the plant nutritional status has also been highlighted for PA1 genes. Under conditions of sulfur-deficiency, the levels of the PA1 mRNAs in developing pea seeds were reduced, without major changes in the transcription rates of the corresponding genes [4,38]. Thus, a post-transcriptional mechanism is responsible for these variations. Indeed, it was later confirmed that the PA1 mRNA level was effectively lowered in the leaves of transgenic tobacco grown under sulfur-deficient conditions [40]. To establish which regions of the gene were involved in the process of sulfur sensitivity, the expression level of a reporter gene supplemented with different sets of PA1 gene sequences (e.g., intron) was measured in transgenic tobacco under different conditions of sulfur supply. These experiments have shown that the intron processing was not the post-translational event affected by sulfur nutritional status but instead it was two elements that determined the sulfur responsiveness of the PA1 gene: one was located in the 3’ region between 189 and 323 bp downstream of the PA1 stop codon while the second one, not clearly identified, was within the coding sequence. The mechanism involved in the degradation of the PA1 mRNA during sulfur deficiency has not yet been identified and several models (e.g., mRNA stability) have been proposed [40]. 

### 5.2. The Diversity of the PA1b Peptides

**Figure 3:**
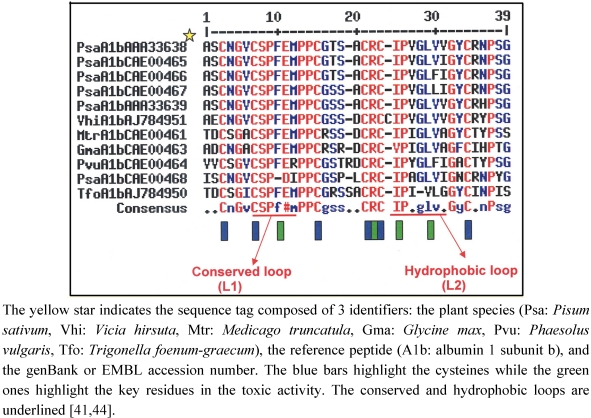
Alignment of PA1b amino-sequences from different Fabaceae.

The *diversity* of PA1b peptides within the same species was initially suggested by the work of Higgins *et al.* [xref^4^] which pinpointed four functional genes that were present in the pea genome and expressed in pea cotyledons. To date, nine peptidic isoforms of PA1b have been isolated and biochemically characterized in the garden pea [3,4,29,41,42,43], indicating that these peptides belong to a multigenic family whose members have diverged slightly (Figure 3). Almost all the forms should have retained their insecticidal properties [42] since amino-acid variations are not located inside key positions for the maintenance of toxic activity (see Section 6).

Research and identification of PA1b homologs in Fabaceae seeds were carried out by developing an approach combining three complementary methods: (1) the molecular one consisted of cloning PA1 homologous genomic sequences; (2) the biological method used a PA1b-specific bioassay, defined by a differential toxicity between a susceptible or a resistant strain of the rice weevil, *S. oryzae* (see Section 3.1), the test being first performed on whole seeds, for a preliminary screening, and then on seeds extracted with different solvants; (3) the biochemical one included the capacity of the extract to compete as a ligand with radiolabeled pea PA1b in its binding to susceptible insect gut membranes, and extracts were also analyzed by HPLC and mass-spectrometry. In this way, new albumin 1 genes, and their associated products, were characterized in the soybean *Glycin max* and in the bean *Phaseolus vulgaris* whereas in alfalfa *Medicago truncatula* no peptide was biochemically detected, despite the presence of high insecticidal activity and of homologous genes [41]. 

The relevance of the approach described above led to a broader study on 88 additional species scattered amongst the three subfamilies (Caesalpinioideae, Mimosoideae and Papilionoideae) of the Fabaceae [44]. 19 PA1 like genes were characterized from numerous Papilionoideae, but not from Caesalpinioideae or Mimosoideae. The alignment of deduced peptide sequences showed that some amino-acids were particularly well conserved at the structural level; cysteine (6), prolines (5) and some glycines (5), as well as the charged arginine in position 21 (as numbered from the PA1b sequence) ([44]; Figure 3; see also Section 5 in this review). The sequences of these homologues also revealed a two loop peptide sequence highly conserved among Legumes (loop L1, residues 7-15 CSPFE) and a less conserved loop (loop L2, residues 23-31) where the hydrophobic property is conserved (Figure 3)[41,44].These results indicate the importance of these conserved portions (loops L1 and L2) for toxicity.

Thus, to date, in plants the PA1b homologous peptides seem to be strictly legume specific, strongly suggesting that this peptide family is an important line of seed defense against insects from the Fabaceae family. 

## 6. The PA1b Structure/Function Relationship

**Figure 4:**
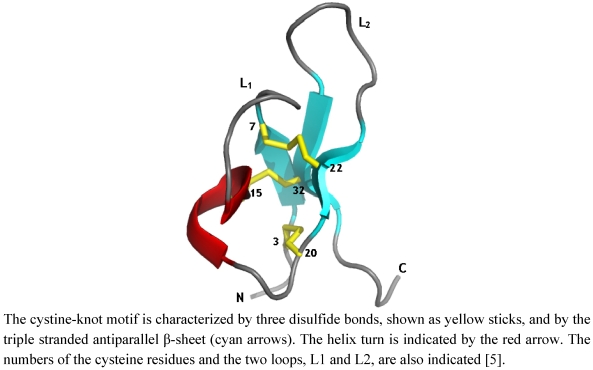
Ribbon representation of PA1b 3-D structure.

The three-dimensional structure of PA1b and the pairing of the disulfide bonds have been established by NMR and molecular modeling [5]. The structure of PA1b displays all the characteristics of the inhibitor cystine-knot (ICK) fold (knottin family), including a triple-stranded antiparallel β-sheet and the cystine-knot motif of disulfide bridges (Figure 4). This structural motif, initially reported for trypsin inhibitors from cucurbits [45,46], is also found in a variety of proteins from plants, fungi, crabs, cone snails and spiders, exhibiting various biological activities [47,48] including ion channel inhibitors, protease inhibitors, antimicrobial peptides and peptides with sweet-taste suppressing effects. In addition, a series of macrocyclic peptides (called cyclotides) from plants, with many biological activities including insecticidal, antimicrobial, hemolytic and cytotoxic activities [49] (such as kalata B1 [50], the first cyclotide discovered), also display a knottin fold. This compact and well-organized scaffold, although not related to a common biological activity, confers to all these molecules a remarkable stability and a high resistance to protease. The specificity of activity presumably results from the type of residues and from their arrangement on the scaffold formed by the ICK motif. 

**Figure 5:**
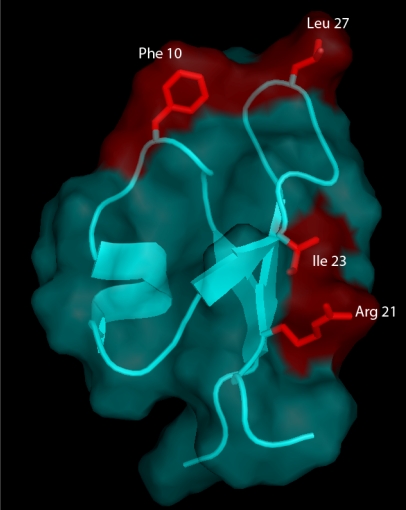
The three-dimensional structure of PA1b and its molecular surface (in cyan) showing the residues (in red) that are essential for receptor binding and insecticidal activity of PA1b [33].

A recent chemical mutagenesis study provides insights into the molecular requirements for the insecticidal activity of PA1b [33]. In this work, the influence of individual residues on the structure and bioactivity of PA1b was investigated. A group of 13 mutants was selected from previous bioinformatic [44] and structural [5] studies which had revealed the importance of residues, located in or around loops L1 and L2, in terms of sequence conservation and/or definition (Figure 4). The thirteen PA1b mutants were chemically synthesized [51] such that the residues involved in the definition of PA1b amphiphilic and electrostatic characteristics were individually replaced with an alanine. The three-dimensional structure of PA1b was outstandingly tolerant of any modifications. Remarkably, receptor binding and insecticidal activities were both dependent on common well-defined clusters of residues located on one single face of the toxin, with Phe-10, Arg-21, Ile-23, and Leu-27 being key residues of the binding interaction (Figure 5). 

Leginsulin (a soybean PA1b isoform) has been shown to be a ligand for the 43-kDa protein in Legumes that control cell proliferation and differentiation [32]. PA1b shares almost 60 percent of sequence identity and slightly more than 80 percent of similarity with Leginsulin [33]. In terms of 3D-structure, the RMS (root mean square) deviation between the backbone heavy atom coordinates of PA1b and leginsulin is very low: 1.5 Å [33]. However, despite these sequence and structure similarities, the residues involved in the binding of PA1b and of leginsulin [32] to their respective membrane targets are different and do not align. Their only common feature is hydrophobicity, except for R21 in the case of PA1b. Finally, the topological arrangement of each set of critical residues, located on opposite faces of the molecules, suggests a different receptor binding mode [33].

As far as we know, only a few cystine-knot proteins have insecticidal activity. They include insecticidal plant cyclotides [52,53,54] and a knottin, *Amaranthus* α-amylase inhibitor (AAI), found in the seeds of *A. hypocondriacus* [55]. The insecticidal activity of AAI relies on a specific inhibition of the α-amylase of insect larvae, such as *Tribolium castaneum*. Concerning cyclotides, it has been postulated that their bioactivities appear to be associated with membrane binding [20]. Until now, PA1b is the only insecticidal knottin acting *via* a membrane protein-based receptor.

## 7. The Expression of PA1 in a Heterologous System

Due to the potential of the PA1b peptide for pest control in sustainable agriculture, the production of the toxin in a heterologous system is interesting both for production on an industrial scale and also for the study and analysis of the peptide at the research level. Indeed, numerous isoforms of PA1b exist, even in one plant (for example, at least nine isoforms are present in the pea), but with sequences so similar that they are very difficult, even impossible, to separate by biochemical methods. Hence, to explore the function of one specific sequence, production in a heterologous system is required. 

Initial attempts at PA1 proprotein production in a heterologous system were performed by Ealing *et al*. in 1994 [56], in plants of agronomic interest, with the aim of improving the quality of pasture protein for ruminant animal nutrition. Then, transgenic tobacco (*Nicotiana tabacum*) and white clover (*Trifolium repens* L.) plants expressing the native pea albumin 1 gene encoding rumen protected peptides (PA1a and PA1b), rich in sulfur amino-acids, were generated by *Agrobacterium*-mediated technology. The PA1 gene was under the control of the ubiquitous 35S CaMV promoter. These assays were not really conclusive since, despite the production of high levels of PA1 mRNA, the corresponding proprotein was barely detectable. Nevertheless it was apparently processed correctly in its two by-products (PA1b subunit: 4 kDa and PA1a subunit: 6 kDa) which were detected in minute amounts, or not at all, as a function of the target tissue (leaf, root, petiole *etc.*) [56].

More recently, in order to protect stored cereals against pests, rice was transformed either with the PA1 genomic sequence or with the full length or partial cDNA under the control of the constitutive maize ubiquitin promoter. Transformed lines were obtained, and it was shown that the seeds of rice plants contain a native PA1b (from analysis by mass spectrometry, by measuring the affinity binding site and by immuno-localization). Transformation with the cDNA corresponding only to the PA1b sequence leads to transgenic lines in which the peptide is not detectable, although PA1b mRNA was expressed. Rice is the first heterologous expression system suitable both for production and for correct folding of the peptide [57].

In addition to the creation of genetically modified plants, the need for a secreted and easy to purify recombinant peptide has led to the development of assays in the bacterial host *E. coli* (a specific mutant strain able to form disulfide bridges) and in the yeast *P. pastoris*. In both cases, organisms were initially transformed with the cDNA restricted to the PA1b part. Despite the presence of the three intramolecular disulfide bonds, the PA1b recombinant peptide was detected in very low quantities and in a non-folded and biologically inactive form [58,59]. However, transformation with the complete PA1 cDNA has not been performed. Recently it has been hypothesized that PA1a plays a role in the acquisition of the PA1b conformation. As a chaperonin protein it could help in the transport and correct folding of PA1b, thus ensuring the biological activity of the toxin. This hypothesis is supported by the fact that in *E. coli*, and from the entire PA1 cDNA, the production of leginsulin (the soybean PA1b homolog) in its active form has been successfully achieved and it has retained its ability to bind with its plant receptor [32].

## 8. Conclusion and Perspectives

Among the insects potentially controllable by the application of PA1b, some have a very important economic or health impact. To cite just two particularly striking examples, grain weevils, which are responsible for losses approaching 25% worldwide (and up to 40% in tropical countries), and the mosquito, for which population control leads to the usual problems associated with chemical pesticides. On a smaller scale, many other applications are possible for specific problems: the development of poison baits (cockroaches, ants); the development of biological insecticides for private gardens; and the protection of wheat flour, as PA1b could be used in organic farming. 

However, the most promising application of PA1b is in the development of transgenic plants (cereals, or others). It is already established that the transformation and production of native PA1b is feasible in rice. However, the quantity of toxin produced may be a problem, and PA1b is acting at relatively high concentration. One way to overcome this difficulty is to enhance the toxicity of the peptide. Using current knowledge of the structure/function relationship, and given the biodiversity of PA1bs amongst Legumes, it would be of great interest to develop the best PA1b sequence possible in order to increase its potentiality.

## References

[1] Bednarek P., Osbourn A. (2009). Plant-microbe interactions: Chemical diversity in plant defense.. Science.

[2] Delobel B., Grenier A.M. (1993). Effect of noncereal food on cereal weevils and tamarind pod weevil (coleoptera, curculionidae). J. Stored Prod. Res..

[3] Delobel B., Grenier A.M., Gueguen J., Ferrasson E., Mbaiguinam M. (1998). Utilisation d’un polypeptide dérivé d’une albumine PA1b de légumineuse comme insecticide.

[4] Higgins T.J., Chandler P.M., Randall P.J., Spencer D., Beach L.R., Blagrove R.J., Kortt A.A., Inglis A.S. (1986). Gene structure, protein structure, and regulation of the synthesis of a sulfur-rich protein in pea seed. J. Biol. Chem..

[5] Jouvensal L., Quillien L., Ferrasson E., Rahbe Y., Gueguen J., Vovelle F. (2003). PA1b, an insecticidal protein extracted from pea seeds (*Pisum sativum*): 1H-2-D NMR study and molecular modeling.. Biochemistry.

[6] Gressent F., Duport G., Rahioui I., Pauchet Y., Bolland P., Specty O., Rahbe Y. (2007). Biological activity and binding site characteristics of the PA1b entomotoxin on insects from different orders.. J. Insect Sci..

[7] Hancock K.R., Ealing P.M., White D.W. (1994). Identification of sulphur-rich proteins which resist rumen degradation and are hydrolysed rapidly by intestinal proteases.. Br. J. Nutr..

[8] Spencer D., Higgins T.J., Freer M., Dove H., Coombe J.B. (1988). Monitoring the fate of dietary proteins in rumen fluid using gel electrophoresis.. Br. J. Nutr..

[9] Desprès L. Université J.

[10] Royer, C.; Rahioui, I..

[11] Rahioui I., Laugier C., Balmand S., Da Silva P., Rahbe Y., Gressent F. (2007). Toxicity, binding and internalization of the pea-A1b entomotoxin in Sf9 cells. Biochimie.

[12] Gressent F., Rahioui I., Rahbe Y. (2003). Characterization of a high-affinity binding site for the pea albumin 1b entomotoxin in the weevil *Sitophilus*.. Eur. J. Biochem..

[13] Eyraud V.

[14] Carre-Pierrat M.

[15] Yamazaki T., Takaoka M., Katoh E., Hanada K., Sakita M., Sakata K., Nishiuchi Y., Hirano H. (2003). A possible physiological function and the tertiary structure of a 4-kDa peptide in Legumes.. Eur. J. Biochem..

[16] Dun X.P., Wang J.H., Chen L., Lu J., Li F.F., Zhao Y.Y., Cederlund E., Bryzgalova G., Efendic S., Jornvall H. (2007). Activity of the plant peptide aglycin in mammalian systems.. FEBS J..

[17] Franco O.L. (2011). Peptide promiscuity: An evolutionary concept for plant defense.. FEBS Lett..

[18] Carvalho Ade O., Gomes V.M. (2009). Plant defensins-Prospects for the biological functions and biotechnological properties.. Peptides.

[19] Craik D.J., Cemazar M., Daly N.L. (2007). The chemistry and biology of cyclotides.. Curr. Opin. Drug Discov. Dev..

[20] Daly N.L., Craik D.J. (2011). Bioactive cystine knot proteins.. Curr. Opin. Chem. Biol..

[21] Grenier A.M., Mbaiguinam M., Delobel B. (1997). Genetical analysis of the ability of the rice weevil Sitophilus oryzae (Coleoptera, Curculionidae) to breed on split peas. Heredity.

[22] Norton R.S., Pallaghy P.K. (1998). The cystine knot structure of ion channel toxins and related polypeptides.. Toxicon.

[23] Chouabe C., Eyraud V., da Silva P., Rahioui I., Royer C., Soulage S., Bonvallet R., Huss M., Gressent F. (2011). New mode of action for a knottin protein bioinsecticide: Pea Albumin 1 subunit b (PA1b) is the first peptidic inhibitor of V-ATPase.. J. Biol. Chem..

[24] Muench S.P., Huss M., Song C.F., Phillips C., Wieczorek H., Trinick J., Harrison M.A. (2009). Cryo-electron microscopy of the vacuolar ATPase motor reveals its mechanical and regulatory complexity.. J. Mol. Biol..

[25] Wieczorek H., Beyenbach K.W., Huss M., Vitavska O. (2009). Vacuolar-type proton pumps in insect epithelia.. J. Exp. Biol..

[26] Wieczorek H., Brown D., Grinstein S., Ehrenfeld J., Harvey W. (1999). Animal plasma membrane energization by proton-motive V-ATPases.. Bioessays.

[27] Toei M., Saum R., Forgac M. (2010). Regulation and isoform function of the V-ATPases.. Biochemistry.

[28] Hanada K., Hirano H. (2004). Interaction of a 43-kDa receptor-like protein with a 4-kDa hormone-like peptide in soybeans.. Biochemistry.

[29] Watanabe Y., Barbashov S.F., Komatsu S., Hemmings A.M., Miyagi M., Tsunasawa S., Hirano H. (1994). A peptide that stimulates phosphorylation of the plant insulin-binding protein-Isolation, Primary structure and cdna cloning. Eur. J. Biochem..

[30] Shang C., Sassa H., Hirano H. (2005). The role of glycosylation in the function of a 48-kDa glycoprotein from carrot.. Biochem. Biophys. Res. Commun..

[31] Shang C., Shibahara T., Hanada K., Iwafune Y., Hirano H. (2004). Mass spectrometric analysis of posttranslational modifications of a carrot extracellular glycoprotein.. Biochemistry.

[32] Hanada K., Nishiuchi Y., Hirano H. (2003). Amino acid residues on the surface of soybean 4-kDa peptide involved in the interaction with its binding protein.. Eur. J. Biochem..

[33] Da Silva P., Rahioui I., Laugier C., Jouvensal L., Meudal H., Chouabe C., Delmas A.F., Gressent F. (2010). Molecular requirements for the insecticidal activity of the plant peptide pea albumin 1 subunit b (PA1b).. J. Biol. Chem..

[34] Dun X.P., Li F.F., Wang J.H., Chen Z.W. (2008). The effect of pea albumin 1F on glucose metabolism in mice.. Peptides.

[35] Hu Z., Dun X., Zhang M., Zhu H., Xie L., Wu Z., Chen Z., Xu T. (2007). PA1b, a plant peptide, induces intracellular [Ca^2+^] increase via Ca^2+^ influx through the L-type Ca^2+^ channel and triggers secretion in pancreatic beta cell. Sci. China C Life Sci..

[36] Graham M.A., Silverstein K.A., Cannon S.B., VandenBosch K.A. (2004). Computational identification and characterization of novel genes from Legumes.. Plant Physiol..

[37] Silverstein K.A., Moskal W.A., Wu H.C., Underwood B.A., Graham M.A., Town C.D., VandenBosch K.A. (2007). Small cysteine-rich peptides resembling antimicrobial peptides have been under-predicted in plants. Plant J..

[38] Chandler P.M., Spencer D., Randall P.J., Higgins T.J. (1984). Influence of sulfur nutrition on developmental patterns of some major pea seed proteins and their mRNAs.. Plant Physiol..

[39] Ilgoutz S.C., Knittel N., Lin J.M., Sterle S., Gayler K.R. (1997). Transcription of genes for conglutin gamma and a leginsulin-like protein in narrow-leafed lupin.. Plant Mol. Biol..

[40] Morton R.L., Ellery A.J., Higgins T.J. (1998). Downstream elements from the pea albumin 1 gene confer sulfur responsiveness on a reporter gene.. Mol. Gen. Genet..

[41] Louis S., Delobel B., Gressent F., Rahioui I., Quillien L., Vallier A., Rahbe Y. (2004). Molecular and biological screening for insect-toxic seed albumins from four legume species.. Plant Sci..

[42] Taylor W.G., Fields P.G., Elder J.L. (2004). Insecticidal components from field pea extracts: Isolation and separation of peptide mixtures related to pea albumin 1b.. J. Agric. Food Chem..

[43] Taylor W.G., Sutherland D.H., Olson D.J., Ross A.R., Fields P.G. (2004). Insecticidal components from field pea extracts: Sequences of some variants of pea albumin 1b.. J. Agric. Food Chem..

[44] Louis S., Delobel B., Gressent F., Duport G., Diol O., Rahioui I., Charles H., Rahbe Y. (2007). Broad screening of the legume family for variability in seed insecticidal activities and for the occurrence of the A1b-like knottin peptide entomotoxins.. Phytochemistry.

[45] Heitz A., Chiche L., Le-Nguyen D., Castro B. (1989). 1H 2D NMR and distance geometry study of the folding of *Ecballium elaterium* trypsin inhibitor, a member of the squash inhibitors family.. Biochemistry.

[46] Holak T.A., Gondol D., Otlewski J., Wilusz T. (1989). Determination of the complete three-dimensional structure of the trypsin inhibitor from squash seeds in aqueous solution by nuclear magnetic resonance and a combination of distance geometry and dynamical simulated annealing.. J. Mol. Biol..

[47] Daly N.L., Craik D.J. (2011). Bioactive cystine knot proteins.. Curr. Opin. Chem. Biol..

[48] Kolmar H. (2009). Biological diversity and therapeutic potential of natural and engineered cystine knot miniproteins.. Curr. Opin. Pharmacol..

[49] Ireland D.C., Clark R.J., Daly N.L., Craik D.J. (2010). Isolation, sequencing, and structure-activity relationships of cyclotide. J. Nat. Prod..

[50] Saether O., Craik D.J., Campbell I.D., Sletten K., Juul J., Norman D.G. (1995). Elucidation of the primary and three-dimensional structure of the uterotonic polypeptide kalata B1.. Biochemistry.

[51] Da Silva P., Strzepa A., Jouvensal L., Rahioui I., Gressent F., Delmas A.F. (2009). A folded and functional synthetic PA1b: An interlocked entomotoxic miniprotein.. Biopolymers.

[52] Barbeta B.L., Marshall A.T., Gillon A.D., Craik D.J., Anderson M.A. (2008). Plant cyclotides disrupt epithelial cells in the midgut of lepidopteran larvae.. Proc. Natl. Acad. Sci. USA.

[53] Jennings C., West J., Waine C., Craik D., Anderson M. (2001). Biosynthesis and insecticidal properties of plant cyclotides: The cyclic knotted proteins from *Oldenlandia affinis*.. Proc. Natl. Acad. Sci. USA.

[54] Jennings C.V., Rosengren K.J., Daly N.L., Plan M., Stevens J., Scanlon M.J., Waine C., Norman D.G., Anderson M.A., Craik D.J. (2005). Isolation, solution structure, and insecticidal activity of kalata B2, a circular protein with a twist: Do Mobius strips exist in nature?. Biochemistr.

[55] Chagollalopez A., Blancolabra A., Patthy A., Sanchez R., Pongor S. (1994). A novel alpha-amylase inhibitor from Amaranth (*Amaranthus hypocondriacus*) seeds.. J. Biol. Chem..

[56] Ealing P.M., Hancock K.R., White D.W. (1994). Expression of the pea albumin 1 gene in transgenic white clover and tobacco.. Transgenic Res..

[57] Petit J. (2006). Etude Structure/Fonction d’une Albumine Entomotoxique de Type A1b du Pois Chez le riz: Application à la Protection Contre le Ravageur *Sitophilus Oryzae*. Ph.D. Dissertation.

[58] Louis S. (2004). Diversité Structurale et d’activité Biologique des Albumines Entomotoxiques de Type A1b des Graines de Légumineuses. Ph.D. Dissertation. http://docinsa.insa-lyon.fr/these/pont.php?&amp;id=louis.

[59] Rahbé Y., Louis S.

